# Human–Robot Interaction and Sexbots: A Systematic Literature Review

**DOI:** 10.3390/s21010216

**Published:** 2020-12-31

**Authors:** Carina Soledad González-González, Rosa María Gil-Iranzo, Patricia Paderewski-Rodríguez

**Affiliations:** 1 Departamento de Ingeniería Informática y de Sistemas, Escuela de Ingeniería y Tecnología, Universidad de La Laguna, 38204 La Laguna, Spain; 2 Departamento de Informática e Ingeniería Industrial, Escuela Politécnica Superior, Universitat de Lleida, 25001 LLeida, Spain; rgil@diei.udl.cat; 3 Departmento de Lenguajes y Sistemas Informáticos, Escuela Técnica Superior de Ingenierías Informática y de Telecomunicación, Universidad de Granada, 18071 Granada, Spain; patricia@ugr.es

**Keywords:** sexual robots, ethics, gender, social robots, human–robot interaction, sexbots

## Abstract

At present, sexual robots have become a new paradigm of social robots. In this paper, we developed a systematic literature review about sexual robots (sexbots). To do this, we used the Scopus and WoS databases to answer different research questions regarding the design, interaction, and gender and ethical approaches from 1980 until 2020. In our review, we found a male bias in this discipline, and in recent years, articles have shown that user opinion has become more relevant. Some insights and recommendations on gender and ethics in designing sexual robots were also made.

## 1. Introduction

Recently, the area of human–robot interaction (HRI) [1], particularly in relation to sexual robots, has begun to attract interest with regard to various social issues, such as emotions, ethics, philosophy, and psychology. These new relationships between sexual robots and humans have also awakened the interest of the media [2], the industry, and the maker world, since with a 3D printer, it is already possible to create a sexual robot [3]. Society has begun to consider the idea of having sex with robots, and there is the belief that this will be normal in the future [4]. Although there is still no scientific evidence of its therapeutic benefits, many think it can help treat sexual dysfunctions or even help decrease women’s sexual exploitation. Like sex toys, some experts consider sexual robots (or sexbots) to have potential in being the future of sex relationships [2]. 

However, there are many unanswered questions about the relationships between these types of social robots and people, i.e., about their safety, about how they affect the “psychological aspects”, about the legal regulations for their use, as, for example, in the case of child sex robots. Therefore, in this paper, we will try to answer the following questions:

RQ1: How are sexbots designed?
RQ2. How do sexbots interact with humans?
RQ3: What gender and ethical issues are related to the design and use of sexbots?

Regarding RQ1, we aim to prove the hypothesis that the design of sexbots is driven not by academic research, but only by the market. This implies that moral issues are not necessarily included. This first question highlights the point that academic research is not used in the design of these sexbots. We wanted to prove that the parameters taken from male preferences are used the most in the design. The second question, RQ2, is about our concern in the kind of relationships that can be established between sexbots and humans. We set out to explore whether these relationships would be healthy. The third question, RQ3, is related to our hypothesis that the design of sexbots does not begin (usually) from the viewpoint of protecting human values.

Thus, in this paper, we conduct a systematic review to answer the mentioned questions, focusing on the ethical and gender approaches about sex with robots. The paper is organized in several different sections. Section 2 presents some current sexbots that are available in the market. Section 3 presents the method followed, and the results are presented in Section 4. Section 5 discusses the obtained results regarding the research questions. Specifically, the process of sexbot design, the interactions with humans, and gender and ethical issues are discussed. Finally, conclusions are presented to provide a roadmap for the designers of sexual robot technology. 

## 2. Background

The realism of the sex dolls has increased over the years. We begin by comparing their evolution from the 17th century, where such dolls were first made of fabric, to those in the 1970s made of latex, silicone, and inflatables, and lastly to the sophisticated models with artificial intelligence today. We can see that the market has changed greatly [5]. In this section, we present some of the current models of sexbots.

To date, there is no consensus on a unique definition of sexual robots, also known as sexbots [6]. We can compare sexbots to sex toys because both are created to have sex with humans [7]. However, sexbots can cause emotions in people, such as love [8]. There are different research areas interested in exploring these new relations among sexbots and humans [9,10,11,12,13,14]. 

**Figure 1:**
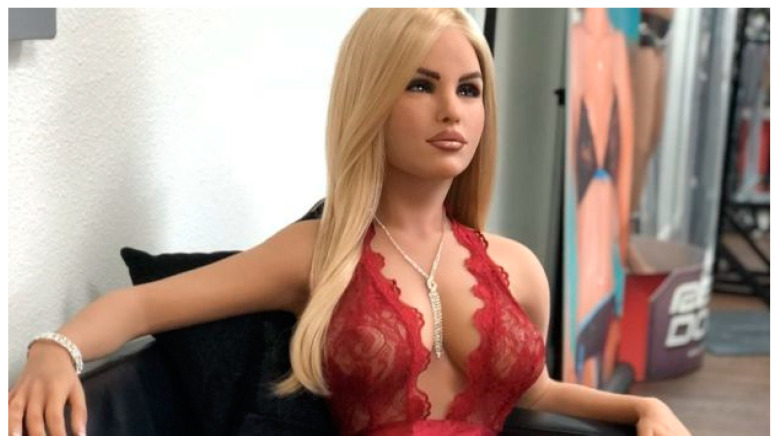
Harmony, marketed as “the perfect companion” with artificial intelligence (Source: http://www.sickchirpse.com/biggest-worry-men-sex-robots/).

**Figure 2:**
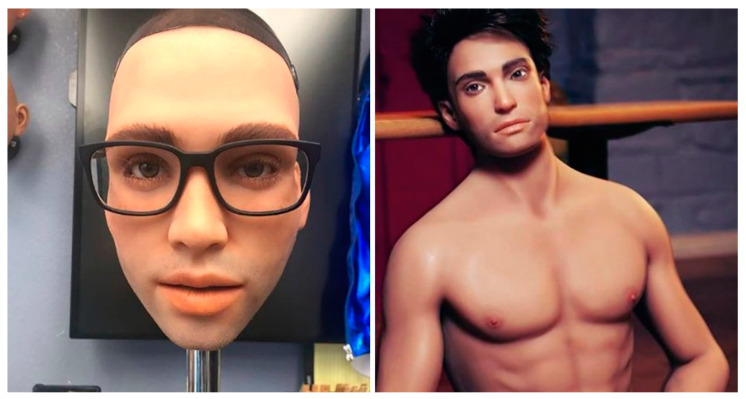
Henry, male version with artificial intelligence (https://realbotix.com/).

Sexbots are a kind of social robots, ones that are personalized, intimate companions. In most cases, sexbots are personalized according to male fantasies. However, both men and women can acquire different models in the market, such as Roxxxy [10], Harmony [11] (Figure 1), or Henry [12] (Figure 2).

Designers of sexbots need to consider the temperature, the psychological and physical issues, among other customizable elements [5]. In addition, some of these sexbots also have a certain intelligence [13]. They can be a reactive machine (i.e., perceive the world and act in consequence), have memory, be based on mind theories, or have self-awareness. In the last case, robots with self-awareness can be considered a sentient robot.

In the next section, we will describe the systematic review that we conducted about sexual robots and their implications for humans.

## 3. Materials and Methods

This article focuses on indexed and peer-reviewed journal articles about sexbots, sexual robots, and sex dolls that were published between 1980 and 2020. A search was conducted in the Web of Science (WoS) and Scopus databases during September 2020 to locate papers on computers, robotics, behavioral sciences, psychology, science, social science, and other topics. An advanced search was conducted using specific terms (i.e., sex AND doll OR sexual robots OR sexbot).

We did several tries in different periods, but no meaningful documents were obtained out of the defined time interval. On the search terms, they are words that are usually related to machines (understood as robots) and sex in interactive systems design, concretely in engineering. The relevant areas for us were related to the designers of this kind of robot, not only in the software but also in the hardware. We found that the article type is the most common way to communicate the latest research with a certain guarantee of scientific quality (peer-reviewed) for the type of papers we selected.

The three authors in this study independently completed an inclusion/exclusion checklist to ensure the systematic review’s reliability while screening the titles, keywords, and abstracts of the primary search. A qualitative analysis was conducted using a consensus agreement method to resolve any differences. 

The inclusion criteria were the following: The type of document must be an article, with English as the chosen language; the period of publication must be between 1980 and 2020, the research topics of the articles must be on either computers, robotics, behavioral sciences, psychology, science, social science, or any other related topics. The exclusion criteria were the following: The study could not be retrieved; the type of article contains opinions or is editorial in type; the article is not related to robots; and the article is neither in English nor in Spanish. The articles were then categorized into three categories: (a) articles excluded due to the exclusion criteria being met; (b) articles excluded due to the inclusion criteria being only partially met with sexbots or robotics; and (c) articles included due to the inclusion criteria being fully met.

We made our analysis based on IEEE P7008—the Standard for Ethically Driven Nudging for Robotic, Intelligent, and Autonomous Systems. Sponsored by the IEEE Robotics and Automation Society, IEEE P7008 delineates the concepts, functions, and benefits necessary to establish and ensure ethically driven methodologies for the design of robotic, intelligent, and autonomous systems following worldwide ethics and moral theories, with an emphasis on aligning the ethics and engineering communities to understand how to pragmatically design and implement these systems. However, it is still under development, which is the reason we have not validated it against a standard.

## 4. Results

The search in WoS using the keywords and the period between 1980 and 2020 resulted in 1244 articles. After filtering by the type of document for results where the document is an article, 1002 papers remained. These 1002 papers were then filtered by topics following the inclusion criteria, and this resulted in 536 papers. After screening the titles and keywords, 55 papers remained for the screening of the abstracts. 

**Figure 3:**
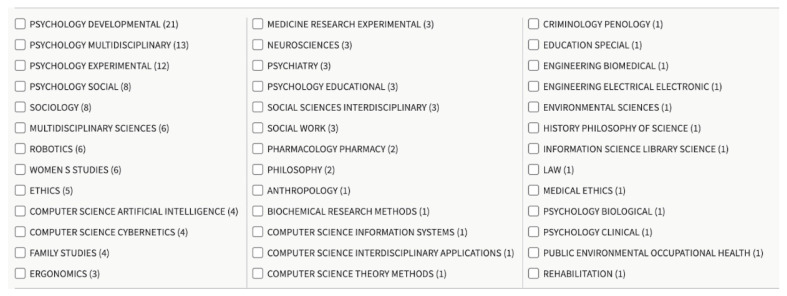
Topics selected in Web of Science (WoS) database.

To have an overview of our database and refine it properly afterward, we performed a series of bibliometric measures on the WoS database with topics that we think are appropriate, as shown in Figure 3. 

We followed the same procedure in the Scopus database with the following results, using the same query (i.e., TITLE-ABS-KEY: sexbot* OR sexual AND robot* OR sex AND doll), which gave us 75 documents. With the filter per year (1980–2020), 73 documents were found. After that, only the articles were selected, which were 54 documents. After the documents were filtered by area, i.e., psychology, social sciences, arts and humanities, computer science, and engineering, 44 documents were selected. The documents that were not related to our investigation were eliminated from the list. After cleaning for duplicates, we added 10 papers to the list of papers to analyze. Finally, we screened the abstracts, as well as 28 papers in the WoS and 9 of Scopus for the full-text analysis. Thus, the total number of papers that met the inclusion criteria for the full-text analysis was 31.

**Figure 4:**
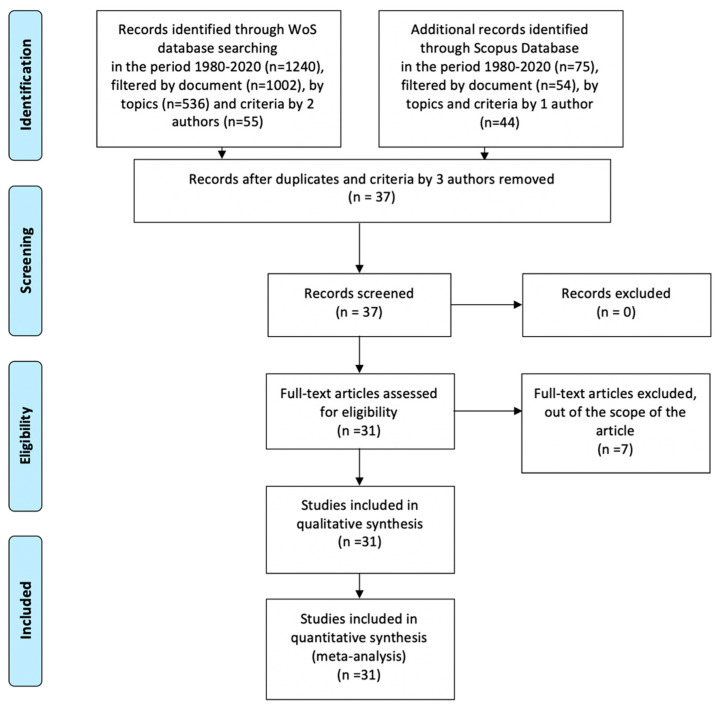
Flow diagram of the study selection process PRISMA (Preferred Reporting Items for Systematic Reviews and Meta-Analyses).

The flow diagram of our study was designed following the recommendations of the Preferred Reporting Items for Systematic Reviews and Meta-Analyses (PRISMA [15]) (Figure 4).

**Figure 5:**
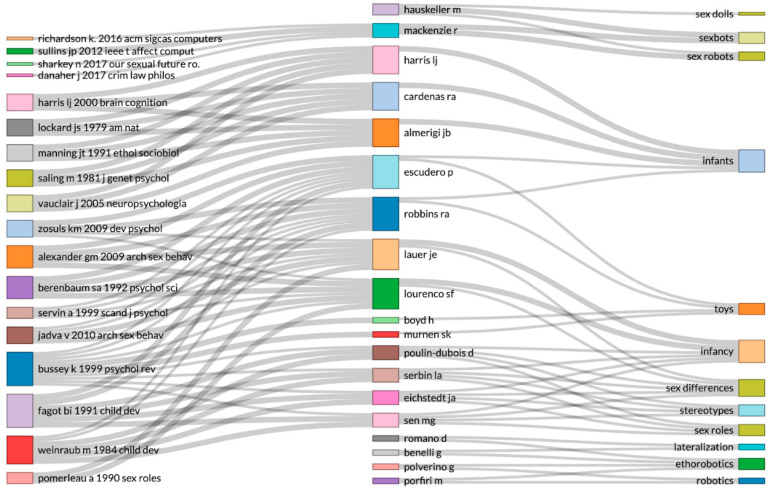
Three-plot diagram relating the references, authors, and topics. This diagram also shows who the authors were that built a concrete term.

In trying to know which authors influence the topics we are interested in, we created a 3-plot diagram in order to draw a relation among the references, authors, and topics (Figure 5). This figure allowed us to find the connection between which authors made the knowledge basis and how journals elaborate the semantics. The words became the metadata with which we could use to search, and they are representative of the topics. 

**Figure 6:**
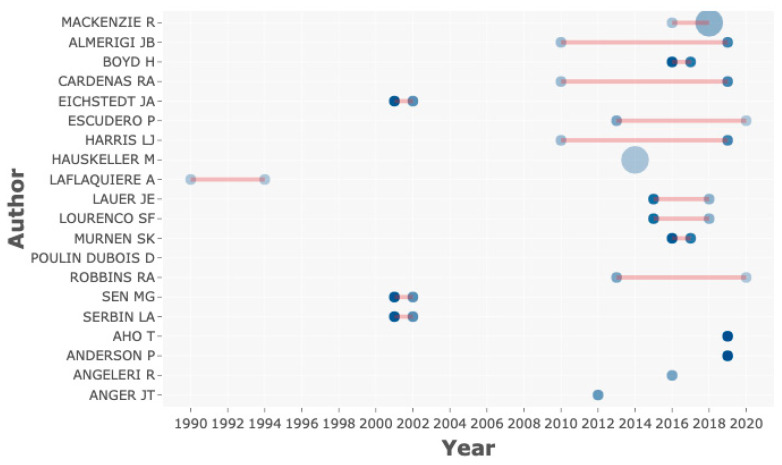
Comparison of the periods of publications according to author search.

Another question that we wanted to know was which time period is the most prolific one. It seems that 2010 marks the beginning of such a period (Figure 6). This kind of graphic allowed us to visualize the density of the activity periods of the chosen authors. Thus, it can be established when the concrete time window is when searching for information. 

**Figure 7:**
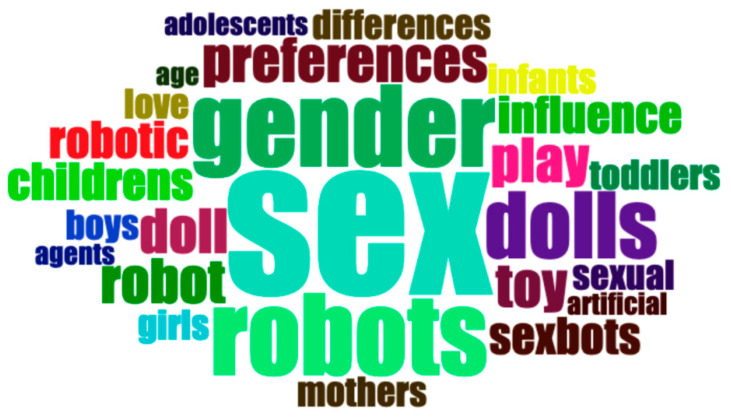
CloudWords with titles of papers found in the review.

We also wondered about the terms associated with our search (i.e., TITLE-ABS-KEY: sexbot* OR sexual AND robot* OR sex AND doll), so we constructed some CloudWords with titles, and we found some unexpected topics, such as mothers and children (Figure 7). This technique emphasizes which terms are the most used, the size, and the position fix, which is relevant.

**Figure 8:**
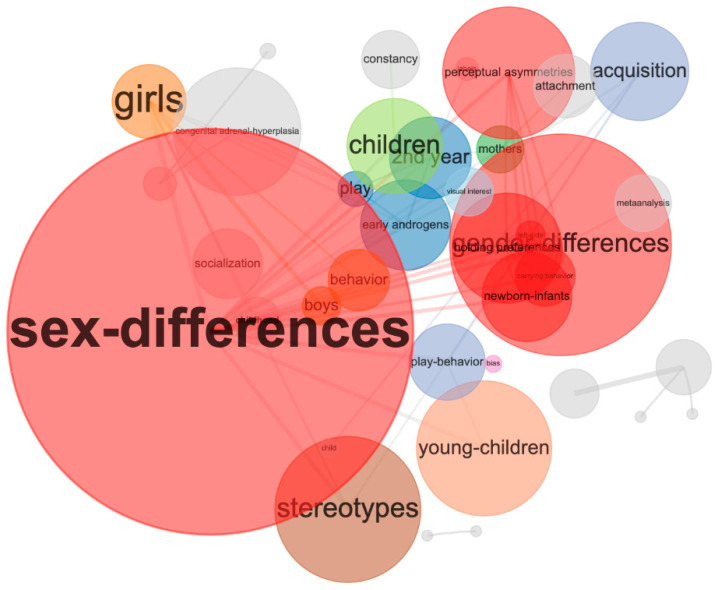
Co-occurrence network of concepts.

We analyzed the co-occurrence network, using edge betweenness as a clustering algorithm, to figure out which concepts were more used and whether they were related (Figure 8). This figure was realized in order for us to look for concepts that we did not consider in our analysis, and they could be interesting concepts to be taken into account in further analysis. Some concepts were not expected, such as play behavior or constancy.

The collaboration network focuses on which countries show the greatest influence in the research of sexbots, and among them were the United Kingdom, the US, and Australia. We could not find any evidence that shows what is happening in Asian countries, and so we found this network to be a bit unrealistic. 

**Figure 9:**
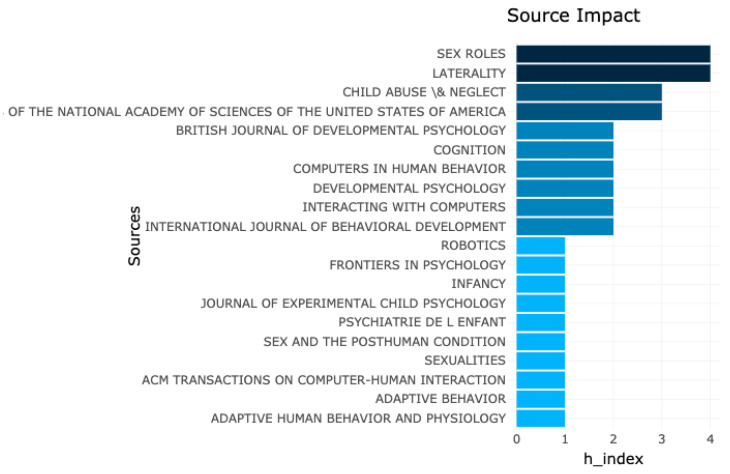
H-index of the sources used in the search.

To measure the source impact, we use the H-index as seen in Figure 9.

We organized and synthesized the final selected studies into three different tables according to the research questions: RQ1 about design approaches (Table 1), RQ2 about interactions (Table 2), and RQ3 about gender and ethical issues (Table 3). When the paper emphasized one aspect over others, we decided to classify it in the option that predominated. In these cases, we chose interaction over the design in the first case and relationships over interactions in the second case.

## 5. Discussion

Design, interaction, and ethics and gender approaches are the three issues we chose to evaluate the literature with. The questions previously mentioned are answered with the literature review in this section.

### 5.1. RQ1: How Are Sexbots Designed?

In a previous work [46], we found that there are different approaches to sex robot design [7,47,48,49,50,51,52,53,54,55,56], of which we can highlight two [48], i.e., the functional ones, which are not based on cognitive functions in designing social robots, and the biological ones, which are based on cognitive models and natural sciences. Furthermore, we can observe other methods coming from biomimetic robotics [47], social robotics [49], and biohybrid neuroprosthetic systems related to biomedical engineering and neuroscience [52,53]. Based on this systematic review, we see that there has been a greater interest in the opinion of human users within the past ten years, more specifically, in men, about how a sex robot should be designed. We hardly found any articles on designing the functionality or the possible biological inspirations that a sex robot’s design may have. Moreover, studies on comparisons between sexbots/sex dolls and human beings deduce exactly which qualities are the most attractive [17,36]. Again, more studies have been done on sex dolls and sex robots for male users [16]. We wonder whether these studies will be decisive when designing sexbots.

Considering the results of this systematic review, we can note that there are sex differences in male and female faces and voices [20]. Males prefer more female voices and stimulus. Some sex toys might also help people with disabilities and people in long-distance relationships (LDR) [25]. In addition, there are guidelines for helping individuals and relational systems make informed choices regarding the participation in technology-based activities [26].

### 5.2. RQ2. How Do Sexbots Interact with Humans?

A sexbot is a social robot [24,54] that can interact with humans through vision (through cameras), voice (through microphones and speakers), touch (through capacitive sensors or contact microphones), cognition, and emotion (through cognitive modeling and behavioral responses, perceiving, and expressing emotions) [54]. 

For example, Samantha [55] is a sexbot who has sensors on her hips, shoulders, vagina, and mouth, and can respond to touch. In addition, it has a sexy or familiar mode, programmed with artificial intelligence, and users decide the context they can interact with it in one way or another. 

Based on our systematic review, the affective aspect can be essential. For instance, in the movie *Guys and Dolls* [24], we find a protagonist in love and married to his robot. Other mechanisms are put in place in the relationships between humans and sex robots apart from sexual ones, as shown in the documentary, such as relationships of control. One male protagonist, who collects different sex robots in his garage, feels he will never control a real woman in the same way as controlling a robot. Another type of relationship is based on the kind being a hobby, that is, as long as an interesting woman does not appear, the user continues to be with a sexbot.

The relationships between human beings (males in this case) and their sex robots also can be very complex, as an analysis in a sex forum demonstrated [21]. The most cited reason was “doll maintenance” for interaction among peers. The study found that peer bonding was the primary factor driving member interaction—a result consistent with studies of pornography forum fan pages in which collectivity and peer approval are paramount in online sexual cultures. Movies such as *2040* [23] fantasizes the sexual relations between human beings and what they called “anabots”, particularly in the scenes that dramatize sex between anabots and humans, allowing the film to comment on the role that technology has.

There is a lack of empirical analyses of doll ownership psychological characteristics or behavioral implications, and no standardized measure of the attitudes towards sex dolls and robots and their owners exists [28]. Moreover, sex therapists and physicians have different opinions about the therapeutic benefits of sex robots [29], although the attitudes toward sex robots as a therapeutic tool were very heterogeneous, depending on gender, age, and occupational differences. Psychologists (in contrast to physicians) were more critical toward the therapeutic use of sex robots. The most frequent use was seen in patients with social anxiety that prevents a sexual life.

One study showed that sex dolls are used for more than just sex [30]. Some owners use dolls to create a sort of embodied intimate fiction. Intimate fantasies are persuasive if they are customizable, which is a characteristic that can be considered in the design of sex robots. In this sense, there is a high prevalence of nonsexual, posthuman companionship dynamics between dolls and their owners [31]. Media representations of intimate human–robot relationships were studied by [32], who found that such representations portray the involved human partner as a disadvantaged man in interpersonal relationships.

Some authors tried to understand the implications of introducing emotions into robotic machinery [19]. In the future, robots can experience emotionally and sexually satisfying partnerships; perhaps the emphasis should be once again placed on humans. The relationship between machines and humans has been studied under the concept of good sex and complete sex, and in this case, their mutual respect is needed [40]. Humor can be another component in the interaction between humans and robots [27]. However, acceptable types of humor should be carefully selected.

### 5.3. RQ3: What Gender and Ethical Issues Are Related to the Design and USE of Sexbots?

We have organized the discussion on this question in two subsections: (a) gender approaches and (b) ethics approaches. Following we present the main related findings.

(a) Gender approaches

In our review, almost no study was found regarding women using male sex robots except in [29]. In this way, both the design and the interaction are biased because there is a male hegemony seen. A female perspective is needed to guarantee gender equity.

Some authors [45] focused on relationships, concretely on jealousy. As we saw in the interactions between humans and robots, as the manufacture of sexbots is perfected, the relationships between humans and these robots will become more complex. Therefore, when the sexbot does not have a single function, and a romantic or emotional relationship appears, gender differences appear between a platonic love robot and a sex robot, in that the robot becomes a partner. In an online study (i.e., a vignette about a sexual robot), females have less favorable views of robots, especially sex robots, compared to men. This means that women place more importance on the fact that their partner got a sex robot rather than a platonic love robot, and females are expected to feel more jealous. Females who read about sex robots reported significantly elevated levels of jealousy, less favorable attitudes, a greater level of dislike, and a greater level of a predicted partner’s dislike. The fear of the unknown, or the partner’s insecurities, is projected onto the partner, causing jealousy to appear.

Media representations of intimate human–robot relationships are also biased. In this sense, some authors [32] explained how media representations of intimate human–robot relationships portray the involved human partner as a disadvantaged man in interpersonal relationships. Therefore, media often portray the involved robot partner as a female humanoid sex robot. Nonfictional media describe intimate human–robot relationships more often in sexual terms because a product or service is offered; fictional media focus more on emotional aspects because this involves a fantasy. Media representations of intimate human–robot relationships reveal stereotypical gender roles, heteronormativity, and a focus on sexual versus emotional intimacy. In all its variants, such as comics, series, books, or movies, science fiction provides habitually hypersexual heroines.

Articles in the past decade focus more on concrete interactions. Some researchers [41] explored gender affordances of conversational agents. Their examination takes a holistic approach in analyzing the application of gender stereotypes to nine chatterbots: six embodied (three male and three female), two disembodied (male and female), and a robot embodiment. Feeling accompanied is not only achieved by physically having an object or someone close. Affectivity again appears as a recurring theme in this field. For this reason, a conversation thought of as an affective interaction is an element that must be taken into consideration. The authors tested the persistence of gender stereotypes in selecting conversation topics (the referential aspect of conversation) and the elicitation of disinhibition and verbal abuse (the relational aspect of conversation). Two main hypotheses were formed, with the first one on a gender-related conversational topic hypothesis. In other words, conversations with female-presenting agents will revolve more around social relations and physical appearance than conversations with male-embodied agents. These can be seen in some everyday examples, such as the conversational agents around us; they usually have a female voice and a woman’s name, such as Alexa, Cortana, or Siri. For the second hypothesis, i.e., the so-called disinhibition hypothesis, the authors expected that conversations with male-presenting agents would more frequently focus on activities, compared to conversations with female-presenting agents. As females are often perceived to have less status and are usually objects of sexual attention, female agents are expected to be the recipients of more disinhibited behavior. In particular, it is expected that female-presenting agents would be the recipients of more sex talk and verbally abusive behaviors than male-presenting agents. It should be noted that this is a risky hypothesis if the sample of users is not biased concerning sexual orientation. They concluded that gender stereotypes tend to affect interaction more at the relational (style) level than at the referential (content) level of conversation. Usually, people attribute negative stereotypes to female-presenting chatterbots more often than male-presenting chatterbots. Female-presenting chatterbots are more often the objects of implicit and explicit sexual attention and swear words. They claimed a more informed analysis of user interactions that considers the full range of user interactions.

Moreover, we consider other groups with different sexual orientations because users follow stereotypical gender patterns when conversing with chatbots presented as either male or female. These gender patterns tended mainly to affect the relational aspect rather than the referential aspect of the conversation. This bias is seen from the investigation. The application of gender stereotypes in the interaction with chatbots often leads to more dismissive attitudes toward women than men.

(b) Ethics approaches

Fortunately, various laws to protect the most disadvantaged individuals, such as children, have appeared in this past decade. Governments should try to protect all these cases that appear, including possibilities that we could not yet imagine.

New crimes under the Sexual Offences Act 2003 (SOA) that address the creation, distribution, and possession of child sex dolls and robots where a real child is involved in their creation has been proposed by [34]. Where sex dolls and robots are fantasy creations, it is argued that different considerations arise, and it is difficult to justify the same range of restrictions. Accordingly, separate SOA offenses are suggested, with exceptions made for self-made artifacts intended solely for private use. In this way, the law adapts to the origin of the sexbot, its conception, and the original idea, separating the fantasy from the physical world’s replica.

One point to always keep in mind is that there is a business chain involved, i.e., the distributor, the seller, the supplier, and the consumer. In this line, we seek to answer questions about what is provided, who consume it, and what they do with it since it can be for their own consumption or redistribution. For laws to be efficient, they must consider all these aspects.

The debate about “seeing the glass half full or half empty” is a common denominator in all these challenges that we must face as a society. Are these products and services an opportunity to help people with sexual or relationship problems [22]? Alternatively, should they be prohibited because they are something that is against nature? An essay about the legal regulations about child sexual robots [42], highlighted the “dark field” problem where the restrictive approach to regulation is the wisest choice because when there are children in the way, they must be protected at all costs so as to not be attacked under any circumstances, which is a point also addressed in [35]. However, all individuals must be protected. In the ethical safeguards into sexual robots, [43] conducted a literature review about the artificial morality in robots/agents because commercializing sex with robots could reinforce existing gender inequalities and sexual objectification. Some issues are considered as the “no consensus”, which depends on the culture, and this was examined by [39], who explored whether it is conceivable, possible, and desirable that humanoid robots should be designed such that they are capable of consenting to sex. They considered the reasons for giving both “no” and “yes” answers to three questions by examining the concept of consent in general, as well as critiques of its adequacy in the domain of sexual ethics, the relationship between consent and free will, and the relationship between consent and consciousness. 

Also, the frame problem where there is an evaluation of the consequences of the acts, was faced by [43], considering that this evaluation involves ethical behavior. This ethical behavior is the object of the evaluation. Another aspect is the ethical boundaries that can be approached by simulating ethical dilemmas. As a particular objective, it was proposed to have contributions to the moral philosophy, assuming that perhaps some traditional theories should be challenged to codify ethics. 

On the positive side, in their ethical and social implications of translating embodied AI applications into mental health care across the fields of psychiatry, psychology, and psychotherapy, [44] conducted a literature review of new modes of treatment, opportunities to engage hard-to-reach populations, better patient responses, and freeing up time for physicians. A lack of guidance on the development of AI applications, their clinical integration, and health professionals, as well as missing points in ethical and regulatory frameworks, are challenging. From a realistic vision, there is a potential for misuse, including using the technologies to replace established services, thereby potentially exacerbating existing health inequalities. Values such as harm prevention and data ethics issues were also highlighted. 

The point of view on sex robots will need to be clarified [33] and challenged as technology advances towards sex robots with “awareness”. While it may be possible to name a multitude of studies on creating artificial consciousness, it appears that to date, no one has yet formulated an unquestionable definition of consciousness since the existing definitions are speculations and models of how consciousness is believed to operate. The nature of consciousness has been and continues to be studied, but there is no unified explanation on how it can be generated. The debate about whether it can be generated in the distant future is also open for debate. However, in the past decade, articles such as [5] speculating the ethical limits and legal implications of customizable human-like robots, which must be addressed urgently, propose a duty that humans have as creators to safeguard the interests and minimize the suffering of created sentient beings before technological advances preempt this possibility. How we design and customize sexbots and how we treat them matters for us, as well as the future of human/human, human/sexbot, and sexbot/sexbot intimate relations for the sake of achieving harmony between humans and sexbots. Moreover, these questions are part of a broader debate on what ethical duties humans as creators owe the sentient entities they create. Codes of ethical design and flexible regulation that build upon and expand existing ethical codes governing intelligent and autonomous systems to balance and safeguard human interests and the created sentient, self-aware entities must be put in place urgently before technological advances preempt them.

Philosophical essays about the nature of sex robots or their behavior are analyzed in relation to the concepts of life and death [38]. There is a struggle between these two concepts when an inanimate subject comes to play. A revision of traditional philosophical theories supports this relationship.

The distance between a robot and a person causes some authors to find ethics of human nature, such as deontological or consequential ethics, as not adequate to be applied in a hypothetical moral of the machine [37].

## 6. Conclusions

Although society has perceived robots differently in the past, these differences are minimal today, depending on their culture or religion. This paper analyzed the state-of-the-art concerning sex robots, with a focus on the design, interaction, and gender and ethics approach in the last 40 years, by doing an extensive review in Scopus and WoS. As the principal outcomes of this systematic study, we noted the following:

- Some laws are in place with the aim to protect vulnerable individuals, even children.
- There is a real concern about how the interactions between sexbots and human beings will become. Positive and negative consequences appeared in the literature.
- The sexbot design process appears to be oriented by the opinions of human users.
- Design dark patterns must be avoided in designing sexual robots.
- Technology seems to be mature enough to claim a user-centered design.
- Male bias is present in the design, interaction, and even ethics.
- Positive therapeutic uses of sexual robots were found in the literature.
- Sexual robots and dolls are used more than just sex (as companions, friendship, fantasy, etc.).
- Sexual robots are stereotyped, mainly based on female figures (pornography).
- Regarding data related to pedophiles and child-robots, there is currently no evidence of a relationship between the two [44]. However, restrictive regulations on child sex robots are recommended instead of open to experimentation.

Interaction with robots is becoming more realistic, which can affect human perception. Therefore, issues such as ethics and gender must be considered in the design of sexual robots. IEEE initiatives consider ethics in the creation of technology [56], as well as other initiatives [56], which can assist in the design of sexual robots. In addition, new philosophical paradigms appear when we talk about sentient robots. Therefore, both designers and users must be aware of the consequences of the use of sexbots.

## Figures and Tables

**Table 1:** Research question 1 (RQ1) about design approaches, type of study, and outcomes.

Authors	Goals	Keywords	Type of Study	Main Outcomes
Green, MacDorman, and Vasudevan (2008) [16]	To measure human responses to varying facial proportions in people, androids, mechanical-looking robots, and two/three-dimensional characters	Anthropomorphism, attractiveness perception, facial acceptability, interrater agreement, uncanny valley	Survey study	Significant correlations were made between the selection of best proportions and ratings of human likeness and attractiveness.
Norton et al. (1996) [17]	To compare actual proportions of adults’ body shape with dolls (Barbie and Ken)	Social psychology, body shape	Anthropometric study	Unrealistic body proportions of Barbie and Ken were compared to real humans.
Szczuka et al. (2017) [18]	To compare men’s sexual attractiveness of sex robots and women	Sex robot, personality traits, attractiveness, HRI	Survey study	A negative attitude towards robots is the main user characteristic that predicts the attractiveness ratings of sex robots.
Rousi (2018) [19]	To problematize human–robot love and sex relationships	Emotions, robots, sex, infidelity, artificial intelligence	Review essay	Human–robot love and sex relationships are problematized in the eventuality of artificial emotions.
Hou and Ye (2019) [20]	To test out sex differences in preferences for male and female faces and voices	Sex differences facial attractiveness, vocal attractiveness, mate preferences	Computerized test	Men preferred voice recordings and multimodal stimuli of women. Women did not show different attractiveness ratings for the voices of men vs. women.

**Table 2:** Research question 2 (RQ2) about interactions, type of study, and outcomes.

Authors	Goals	Keywords	Type of Study	Main Outcomes
Middle week (2020) [21]	To determine the primary function of a sex doll forum for its users and perceptions of sex robot technology	Hegemonic masculinity, homosociality, human–robot relationships, intimacy sex dolls, sex robots	Qualitative analysis study of a major sex doll forum	Peer bonding was the primary factor driving member interaction. Complex and dynamic homosocial relations characterized men’s online interaction.
Döring et al. (2018) [22]	To review the state of technological development and research regarding sex toys, sex dolls, and sex robots marketed on the Internet	Sex toys, sex dolls, sex robots, sexual products, positive sexuality, sexual pleasure	Review	Positive impact was found in terms of sex education, sexual therapy, sexual counseling, and sexual well-being for interested target groups (people with disabilities, seniors).
James (2017) [23]	To analyze narratives on the sex between anabots and humans	Dystopia	Movie review	The creation of sexual desire as well as the nature of objectification was found.
Burr-Miller (2013) [24]	To study how men have a relationship with real dolls	Heteronormativity, gender performativity, sexuality, iDollators, Guys and Dolls	Essay	Men’s violations of the charmed circle are framed in the documentary due to unrequited heterosexual desires.
Gomes and Wu (2018) [25]	Design a sex toy for people with disabilities and people in long-distance relationships	Brain-computer interface, sex toys, long-distance relationships	Product design	The Neurodildo, a sex toy remotely controlled by brain waves, which is pressure sensitive and has electrical stimulation (e-stim) feedback, was presented.
McArthur and Twist (2017) [26]	To create a design framework for digisexuality	Digisexuality, sexbot, robot, virtual reality, sex therapy	Essay	A framework for understanding the nature of digisexuality and how to approach it is imperative.
Tay et al. (2016) [27]	To study humor as an element of interaction between robots and humans	Social robots, humor, social attraction, facial expression	Experimental study	Humor can be an effective way to enrich interactions between humans and robots, but the acceptable types of humor should be carefully selected.
Harper and Lievesley (2020) [28]	To examine the veracity of the existing psychological, sexological, and legal literature concerning doll ownership	Sex dolls, sexuality, sex robots, sex offending, sexual abuse prevention	Literature review	There is a lack of analyses of the psychological characteristics or behaviors of sex doll owners. No standardized measure of attitudes towards sex dolls and robots exists. It was found that 70% of owners used the robot as a sexual companion, while 30% used it for social companionship/friendship.
Eichenberg, Khamis, and Hübner (2019) [29]	To measure attitudes of sex therapists and physicians toward the therapeutic uses of sex robots	Robotics, sexual health, therapy	Quantitative online survey and a qualitative interview study	Therapists and physicians could recommend sex robots in therapy. Heterogeneous attitudes were found. Psychologists were more critical toward the therapeutic use of sex robots. Patients with social anxiety can benefit from using sex robots.
Su et al. (2019) [30]	To examine how people in the future might relate to robots and similar technologies and agents	Sexuality, intimacy, wellness, care, embodiment, robots, online forums	Qualitative analysis of forums	Sex dolls are used for more than just sex. Fiction and intimate fantasies are more persuasive if they are customizable (sexual robots).
Langcaster-James and Bentley (2018) [31]	To explore motivations and experiences of those who purchase and use sex dolls	Sex doll, sex robots, companionship, posthuman kinship, allodoll	Mixed methods	Some doll owners appeared to establish a rich fantasy life, generating characters and personalities for their dolls and considering what they might think or say.
Döring and Poeschl (2019) [32]	To analyze media representations of intimate human–robot relationships	Human–robot relationships, sex robots, media representation, sexual script theory, gender roles, media content analysis	Content analysis	Media often portray the involved robot partner as a humanoid female sex robot. Nonfictional media describe intimate human–robot relationships more often in sexual terms; fictional media focus more on emotional aspects.

**Table 3:** Research question 3 (RQ3) about gender and ethics, type of study, and outcomes.

Authors	Goals	Keywords	Type of Study	Main Outcomes
Whitby (2008) [33]	To make ethical recommendations for designers	Robot ethics, abusive interaction, ethical design	Essay	Reflections on the sexual-affective effects of uses of sex robots were found. Robots that behave similarly as humans do are precisely the sort of robots most likely to be abused.
Chatterjee (2020) [34]	To explore the debate on whether child sex dolls and robots could and should be caught by the child protection framework	Child sex dolls, child sex robots, criminalization	Essay	New crimes under the Sexual Offences Act 2003 (SOA) were proposed.
Brown et al. (2019) [35]	To analyze the risk and possible negative impacts of the use of child sex dolls	Child sex dolls, crime, criminal justice	Review	Interaction with child sex dolls could increase the likelihood of child sexual abuse and normalize the behavior in the abuser’s mind.
Ángel (2016) [36]	To explore the risks of the hybridization process and simulations	Feminism, gender violence, media, biotechnology, simulations	Analysis	It is essential to incorporate the gender perspective into the technological impact.
Yulianto (2019) [37]	To study the ethical issues and legal regulations of sex robots	Transhumanism, human enhancement, posthuman	Essay	Human-nature ethics, such as deontological or consequentialist ethics, are not suitable for machine morality.
Connor (2015) [38]	To explore the objectification of women through sex dolls	Dolls, women, objectification	Essay	The doll brings together the histories of sexual desire and religion.
Mackenzie (2018) [5]	To debate on sex robots and their social interaction with humans	Sexbot, roboethics, robot law, and right	Review essay	The ethical limits and legal implications of customizable human-like robots must be addressed urgently.
Frank and Nyholm (2017) [39]	To explores the designing of humanoid robots	Sex robots, rape, artificial intelligence, consent, legal community	Review, discussion	The discussion on whether it is conceivable, possible, and desirable that humanoid robots should be designed such that they can consent to sex was explored.
Ess (2017) [40]	To explore love and sex between robots and humans	Social robot, sexuality, love, and sex	Philosophical dissertation, essay	Social robots lack substantial autonomy, genuine emotion, and self-awareness, thereby falling short of what is required by the complete experience of sex to establish experiences of mutual desire, love, and respect.
Brahnam and De Angeli (2012) [41]	To explore gender affordances of conversational agents	Sexuality and HCI, gender, agent abuse, embodied conversational agents, sex stereotypes	Test	The application of gender stereotypes in the interaction with chatterbots often leads to attitudes more dismissive toward women than men.
Danaher (2019) [42]	To debate on regulatory issues about child sexual robots	Legal regulation, child sexual robots, experimentation, restrictions	Essay	The restrictive approach to regulation is the wisest choice.
Headleand, Teahan, and Cenydd (2020) [43]	To explore ethical safeguards into sexual robots as well as artificial morality in robots/agents	Sexbots, artificial sexuality, artificial ethical agent, moral agency	Literature review	Commercializing sex with robots could reinforce existing gender inequalities and sexual objectification. No consensus on what is considered moral between cultures exists. Review also covers ethical boundaries and contributes to moral philosophy.
Fiske, Henningsen, and Buyx (2019) [44]	To explore ethical and social implications of translating embodied AI applications into mental health care	Artificial intelligence, robotics, ethics, psychiatry, psychology, psychotherapy, medicine	Literature review	New modes of treatment and opportunities were presented. A lack of guidance on the development of AI applications was found. Gaps in ethical and regulatory frameworks exists. Harm prevention and data ethics issues were covered.
Nordmo et al. (2020) [45]	To explore relationships and gender differences about platonic love robot or a sex robot	Robot, relationships, jealousy, gender differences, companionship, sex, artificial intelligence	Online study (vignette about a sexual robot)	Females have less positive views of robots, and especially of sex robots, compared to men. People project their feelings about robots onto their partner.

## Data Availability

No new data were created or analyzed in this study. Data sharing is not applicable to this article.
